# Biomechanical evaluation in runners with Achilles tendinopathy

**DOI:** 10.6061/clinics/2021/e2803

**Published:** 2021-05-27

**Authors:** Nathalie Ferrari Bechara Andere, Alexandre Leme Godoy-Santos, Luis Mochizuki, Marcelo Bordalo Rodrigues, Túlio Diniz Fernandes, José Maria Soares-Júnior, Angélica Castilho Alonso, Natália Mariana Silva Luna, Guilherme Carlos Brech, Júlia Maria D’Andréa Greve

**Affiliations:** I Laboratorio de Estudos do Movimento, Instituto de Ortopedia e Traumatologia (IOT), Hospital das Clinicas HCFMUSP, Faculdade de Medicina, Universidade de Sao Paulo, Sao Paulo, SP, BR.; II Departamento de Cirurgia Ortopedica, Instituto de Ortopedia e Traumatologia (IOT), Hospital das Clinicas HCFMUSP, Faculdade de Medicina, Universidade de Sao Paulo, Sao Paulo, SP, BR.; III Laboratorio de Biomecanica, Universidade de Sao Paulo, Sao Paulo, SP, BR.; IV Disciplina de Ginecologia, Departamento de Obstetricia e Ginecologia, Hospital das Clinicas HCFMUSP, Faculdade de Medicina, Universidade de Sao Paulo, São Paulo, SP, BR.; V Programa de Ciencias do Envelhecimento, Universidade Sao Judas Tadeu (USJT), Sao Paulo, SP, BR.

**Keywords:** Tendinopathy, Achilles Tendon, Running, Ground Reaction Force, Biomechanical Phenomena, Muscle Strength

## Abstract

**OBJECTIVES:**

To evaluate the clinical characteristics, ground reaction force (GRF), and function of the plantar muscles and dorsiflexors of the ankle in runners with and without Achilles tendinopathy (AT) and in non-runners.

**METHODS:**

Seventy-two participants (42 men, 30 women; mean age: 37.3±9.9 years) were enrolled in this cross-sectional study and divided into three groups: AT group (ATG, n=24), healthy runners’ group (HRG, n=24), and non-runners’ group (NRG, n=24). Both ankles were evaluated in each group. The American Orthopedic Foot and Ankle Society (AOFAS) Ankle-Hindfoot Scale was used for clinical and functional evaluation. GRF was evaluated using force plates and muscle strength was evaluated using an isokinetic dynamometer.

**RESULTS:**

The AOFAS scores were lower in the ATG. The strike impulse was higher in the ATG than in the HRG and NRG. However, GRF was similar among the groups. The ATG exhibited lower total work at 120°/s speed than the HRG. The peak torque in concentric dorsiflexion was lower in the NRG than in the ATG and HRG. The peak torque and total work in concentric plantar flexion were lower in the NRG than in the ATG. The peak torque and total work in eccentric plantar flexion were lower in the NRG than in the ATG and HRG.

**CONCLUSION:**

Runners with AT showed higher strike impulse, lower muscle strength of the plantar flexors, and higher clinical and functional damage.

## INTRODUCTION

Achilles tendinopathy (AT) is one of the most common overuse injuries in elite and recreational distance runners (1,2). Multiple factors (3-7) are related to AT including intrinsic factors such as poor vascularization, overweight, aging, male sex, height (4,5,7,8), lower limb misalignment, dysfunction and weakness of plantar flexors (3-5,9-14), decreased flexibility, excessive pronation, cavus feet, and lateral instability of the ankle (3,4,10,11,14-16) and extrinsic factors such as old and bad conditions of running shoes, hard surface, conditions related to environment and equipment (8,9,11,14), and training mistakes including those involving the distance, intensity, running rhythm, technique, and fatigue (5,8,9).

AT affects runners, but it is not clear how this condition changes the running technique.Among the spatiotemporal variables, runners with AT may exhibit similar (1) or slower gait speed, shorter stride length, and shorter step length compared to those without AT (17). Ogbonmwan et al. (18) suggested that reduced spatiotemporal gait variables constitute a protective and compensatory mechanism. Among the kinetic variables, Azevedo et al. (1) and McCrory et al. (13) did not find differences in the vertical ground reaction force (vGRF) between healthy runners and runners with AT. Runners with a higher foot impact are at an increased risk of developing lower limb overuse injuries (19,20). Although vGRF indicates the body impact during running, runners with AT do not have a higher vGRF than healthy runners (13). McRoys et al. (13) suggested that peak torque in plantar flexion, touchdown angle, and years of running were the strongest discriminators between runners with Achilles tendinitis and runners who had no history of overuse injury. It is unclear whether healthy runners and runners with AT exhibit any kinetic differences during running. 

Doubts still exist regarding the effect of mechanical factors on the etiopathogenesis and evolution of Achilles tendon injuries in runners (6). Runners with Achilles tendon injuries experience functional loss in sports performance not only due to decrease of strength. Functional losses and changes in the plantar flexor muscles are associated with alterations in ground reaction force (GRF), especially during foot strikes. It is unclear whether runners with AT exhibit a different foot strike pattern compared to healthy runners? Thus, our objective was to evaluate the clinical characteristics, kinetic variables, and the strength of ankle plantar flexors and dorsiflexors in runners with and without AT and in non-runners. Our first hypothesis was that runners with AT would exhibit lower muscle strength than runners without AT. The second hypothesis was that runners with AT would show a different foot strike pattern than the others. We expected that runners with AT would exhibit altered plantar flexor muscles, affecting their foot strike pattern during running.

## METHODS

### Study location and ethical issues

This cross-sectional study was conducted at the Motion Study Laboratory of the Department of Orthopedics and Traumatology, University of São Paulo. Ethical approval was granted by the Ethics Committee of the University of São Paulo (number 0422/11).

### Sample Size

The sample size calculation was based on a previous study (13). An isokinetic variable (peak torque of the plantar flexors) was used to find a difference of 4.3 Nm between the AT group and the healthy runners’ group (HRG). The power of the test was set at 90% (sampling power) with a 5% (two-tailed alpha) level of significance. To meet these conditions, at least 24 subjects were required in each group.

### Subjects

Seventy-two adults (42 men and 30 women) were divided into three groups. The AT group (ATG, n=24) comprised of competitive (professional/elite athletes or those who participated in international competitions) and recreational (individuals running in a nonprofessional or amateur context) runners (21) who had been running at least 20 km/week for 1 year and had suffered an injury within 5 years before the evaluation. The HRG, (n=24) comprised of recreational and competitive runners who had been running at least 20 km/week for 1 year and had not suffered any injury (requiring medical care or stoppage of running) in the last 2 years. The non-runners’ group (NRG, n=24) comprised of non-athletes who did not practice any regular sports or physical activities (less than three times/week). All participants were evaluated using ankle magnetic resonance imaging (MRI). They were also evaluated by an orthopedic physician (foot diseases specialist) who performed a clinical evaluation to diagnose and classify the tendon injury and to verify the absence of other injuries that could constitute the exclusion criteria. The inclusion criteria were as follows: (1) age between 25 and 50 years and (2) absence of neurological, cardiovascular, or cardiorespiratory impairment and/or any mental disturbances or disorders. The specific inclusion criteria for the ATG were: (1) no use of medications in the last 60 days, (2) presence of Achilles tendinopathy (inflammatory process) or tendinosis (degenerative process) with no calcaneal tendon rupture on MRI, and (3) absence of previous lower-limb surgery. The exclusion criteria were the presence of pain or inability to complete any of the tests.

### Procedures

Participants answered a questionnaire about their personal training protocols. They were submitted to a clinical evaluation to analyze their AT. For clinical and functional evaluation, the American Orthopedic Foot and Ankle Society (AOFAS) Ankle-Hindfoot Scale was used followed by evaluation using ankle MRI. The AOFAS scale includes nine items that can be divided into three subscales (pain, function, and alignment). The pain subscale consists of one item with a maximum score of 40 points, which indicates no pain. The function subscale consists of seven items with a maximum score of 50 points, which indicates full function. The alignment subscale consists of one item with a maximum score of 10 points, which indicates good alignment. The maximum score is 100 points, indicating no symptoms or impairment (22).

GRF was measured using two plates (1 kHz sampling frequency, ORC6, AMTI, MA, USA). The participants ran (at 3.0 to 4.0 m/s) on a 10-m sidewalk where these two force plates were mounted right in the center. They performed 10 trials: five for familiarization and five for records. GRF was low-pass filtered with fourth-order Butterworth filter at 100 Hz and normalized by body weight. The maximum vGRF (Fmax), strike impulse (GRF integral during the first 50 ms of contact), and total impulse (GRF integral during the full stance phase). MATLAB scripts (MATLAB 2015; MathWorks, CA, USA) version (8.5) were processed and used to calculate the GRF variables (23).

Isokinetic dynamometry was performed using the Biodex^®^ Multi-Joint System 3 (Biodex Medical; Shirley, NY, USA). The isokinetic dynamometer was calibrated for 30 minutes before starting the tests. The participants underwent this measurement after the running test. Thus, they were already warm. For concentric evaluation of dorsiflexion and concentric and eccentric evaluation of plantar flexion of the ankle joint, the participants were positioned such that they remained seated with the hips in 90° flexion. The biological axis of motion of the ankle joint was aligned with the mechanical axis of the dynamometer and the knee was held at 30° flexion. The rigid plate allowed a 20° range of plantar flexion from the neutral position of the ankle. The participants were held in this position by two thoracic belts, one pelvic belt, Velcro straps on the distal portion of the thigh, and Velcro straps on the metatarsal area in the dorsal region of the foot (24).

All tests were bilateral and standardized and the right lower limb was evaluated first. The subjects performed three submaximal repetitions to familiarize themselves with the equipment, followed by a 60-second rest interval. For data collection, a set of four repetitions at a velocity of 60°/s and another set of 20 repetitions at 120°/s were completed in the concentric-concentric (con-con) mode for both plantar flexion and dorsiflexion and in the concentric-eccentric (con-ecc) mode for plantar flexion. Constant standardized verbal encouragement was provided during the tests to promote maximum effort during contractions (25). The isokinetic variables included the maximum peak torque corrected for body weight (PT/BW) value in % and total work (J).

### Statistical analysis

Shapiro-Wilk test was used to test the normal distribution of the variables. Student’s t-test (parametric distribution) and Mann-Whitney U test (non-parametric distribution) were used to compare the variables between sides. Among the 24 individuals from the ATG, 17 had unilateral injuries and 7 had bilateral injuries. For individuals with unilateral injury, ankles with and without injury were compared. For individuals with bilateral injury, the dominant and the non-dominant sides were compared.

Whenever both the sides exhibited similar results for kinetic and isokinetic variables, only the data from the injured side were analyzed (31 ankles). Since the dominant and the non-dominant sides in the HRG and NRG exhibited similar kinetic and isokinetic results, their results were grouped together with each group containing 48 ankles.

Analysis of variance was used to compare the kinetic and isokinetic variables among the ATG, HRG, and NRG. Bonferroni post-hoc test was used for within-group comparisons. SPSS for Windows, version 15.0 (SPSS Inc., Chicago, IL, USA) was used for the analyses and *p*<0.05 was considered statistically significant.

## RESULTS

Table 1 shows the comparison of baseline characteristics (mean values, standard deviations, and results for testing the hypothesis of equality) among the groups.

Sixteen (67%) runners from the ATG continued training with the same intensity, but presented lower competition performance. Four (16.5%) runners maintained the same training and competition performance and 4 (16.5%) runners showed poor performance during training and competition. Twenty (83%) runners from the ATG experienced pain while running, but they continued running. Two (8%) runners did not experience any pain during running, but experienced it after running. Two (8%) runners stopped running due to pain.

**Figure 1:**
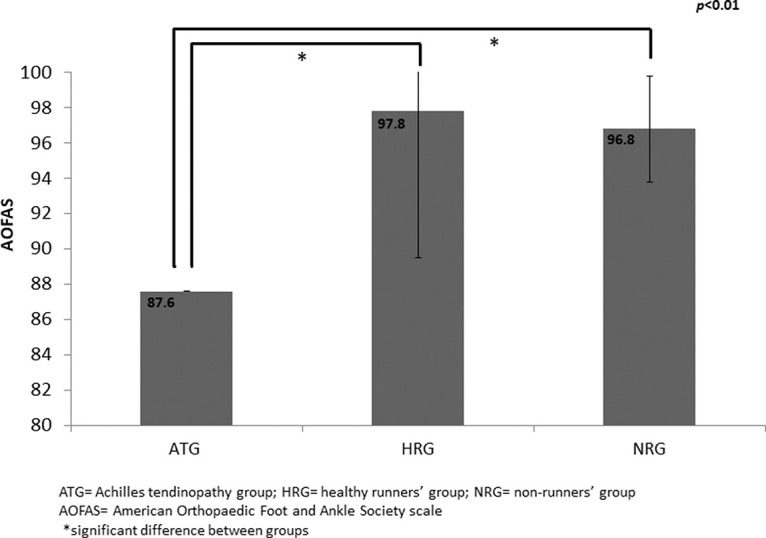
Mean (standard deviation) of American Orthopedic Foot and Ankle Society scale scores in the Achilles tendinopathy group, healthy runners’ group, and non-runners’ group.

The ATG exhibited lower AOFAS score than the HRG and NRG (Figure 1).

The vGRF peak was similar among the ATG, HRG, and NRG. The ATG exhibited a higher impulse in the first 50 ms of contact than the HRG and NRG. The total impulse was similar among the three groups (Table 2).

The isokinetic variables at 60°/s are listed in Table 3. In the con-con mode, there were no difference in the total work of plantar flexors among the groups. The peak torque in the HRG was higher than that in the NRG. In the con-ecc mode, concentric total work of the plantar flexors was higher in the ATG than in the NRG. The eccentric total work in the NRG was lower than that in the ATG and HRG.

The isokinetic variables at 120°/s are listed in Table 4. In the con-con mode, the total work of the plantar flexors in the ATG was lower than that in the HRG. The dorsiflexor peak torque in the NRG was lower than that in the ATG and HRG. In the con-ecc mode, the plantar flexor peak torque and total work were higher in the ATG than in the NRG. The eccentric peak torque and total work in the NRG were lower than those in the ATG and HRG.

## DISCUSSION

In the present study, we compared the results of kinetic analysis (GRF) and muscle strength analysis (isokinetic dynamometer) among runners with AT, runners without AT, and the non-runner control group. Runners in the ATG exhibited the lowest AOFAS score and the highest strike impulse. They also experienced pain, functional loss, and loss of biomechanical alignment. Although most of the injured runners continued running, many of them ran with pain and decreased performance. Worse performance and pain may be associated with lower muscle resistance and fatigue. This in turn causes impairment of long-term resistance, which is essential for distance runners. We observed that healthy runners had stronger dorsiflexor muscles than non-runners.

The maximal vGRF was similar among the ATG, HRG, and NRG. This peak occurred during the propulsion phase and the participants ran at the same speed. Other authors have not found differences in vGRF between runners with and without AT (1,13). However, GRF increased in runners with tibial stress fractures (26) and plantar fasciitis (27). The total impulse was similar among the three groups, as all participants were running at the same speed. McCrory et al. (13) and Azevedo et al. (1) reported similar total impulses between runners with and without AT. These authors did not evaluate the strike impulse.

AT may change the foot strike pattern during running. The ATG exhibited a higher strike impulse than the HRG and NRG. This result supports our hypothesis that runners with AT would exhibit a different foot strike pattern than the other groups. Wyndow et al. (28) found alterations in sural triceps muscle activity in runners with AT, which may impair impact absorption during foot strike. It is possible that plantar flexor eccentric training may improve pain and performance. In military recruits (12), AT leads to a decrease in the force of the plantar flexor muscles. Runners with and without AT had stronger dorsiflexor muscles than the controls. Gymnasts and soccer players also have higher dorsiflexion peak torque, as their sports activities demand high ankle stability (29).

Runners with AT have higher concentric and eccentric total work of the plantar flexors than non-runners. Runners are more physically trained than non-runners due to the higher eccentric activity during running, especially in downhill areas (30). Haglund-Åkerlind and Eriksson (31) found lower eccentric torque at 30°/s, 60°/s, and 120°/s in runners with AT than in runners without injury. Silbernagel et al. (2) observed lower functional capacity in subjects with AT during jumping and strength tests. The Achilles tendon injury did not decrease the muscle performance in the ATG, which was the same as that in the HRG.

Muscle power and resistance are essential for performance in sports and for prevention of injury (2). The plantar flexors exhibited lower total work in the ATG than in the HRG at a speed of 120°/s, corroborating the results reported by O’Neill et al. (32). These data show that endurance and fatigue can contribute to injury. Achilles tendon injury may result in decreased contractile capacity of the musculotendinous unit and increased susceptibility to muscle fatigue (10) with loss of performance over the time course of muscle activity.

Concentric and eccentric peak torque of the plantar flexors was higher in the ATG than in the NRG. The ATG and HRG exhibited similar eccentric torques. Hence, the injury might not have compromised the eccentric action. In contrast, decreased isokinetic plantar flexor strength is a risk factor for AT (33). This result is contrary to our hypothesis that runners with AT would exhibit lower muscle strength than runners without AT. Although multiple extrinsic factors are related to the injury (shoes, surface, environment, equipment, and training mistakes), it is widely known that trained athletes who perform strength training are stronger and faster than resistance athletes and non-trained individuals. Moreover, long-term low-intensity muscle training can modify the muscle profile and help in the treatment and prevention of injury (34).

The interventions aimed at strength training have been reasonably effective in improving pain and disability in individuals with AT (35). In this context, the observed data regarding GRF, force, and muscle resistance can help in improving rehabilitation programs for people with AT, in injury prevention, and in training preparation for competitions based on these specific variables. A better understanding of kinetic and isokinetic force biomechanics discussed in this study would help the professionals involved in studying the factors related to running and their relationship with AT. This in turn would promote better performance, functionality, and quality of life in clinical practice.

Haglund-Åkerlind and Eriksson (31) observed lower eccentric torque values in runners with AT. Eccentric exercises to treat AT are still controversial, but they decrease pain and improve function (10,11,17,23). AT is associated with different biomechanical and functional alterations including tendinopathy-induced muscle weakness and imbalance that modifies the landing pattern.

The comparison of the muscle and running kinetic conditions among the three groups revealed the low morbidity of the injury. There were many differences between the NRG and the two runner groups. The slow evolution of calcaneal tendon injury with progressive loss of function allowed the maintenance of running practice and contributed to the homogenization of the runner groups in this study. This characteristic of a calcaneal tendon injury might be considered a limitation of the present study, as it would require a larger sample size to obtain significant data. Another limitation was the difficulty in collecting running-related data in a laboratory environment, which can inhibit the execution of natural gestures involved in sports.

However, some clinical implications need to be considered. In the present study, the ATG exhibited increased initial impulse and loss of muscle endurance. These functional changes may be related to the development of tendinopathy. If not corrected, the injury could worsen or result in disability. In runners, assessments of muscle condition (performance and balance) and GRF are essential for maintaining biomechanically safe and functional gestures. Difficulties in assessing running as well as multiple factors related to a calcaneal tendon injury and its insidious evolution contribute to controversies regarding the etiopathogenesis, biomechanical aspects, prevention, and treatment of AT, highlighting the need for further studies. The follow-up of the groups of lifelong runners with successive assessments can help clarify these controversies. We observed a difference in the mean age between the NRG and ATG, which was an allocation problem for the participants.

## CONCLUSION

Runners with AT exhibited higher strike impulse, lower plantar flexor strength (resistance), and higher clinical and functional damage. The association between higher strike impulse and lower resistance could be a predisposing and maintaining factor for Achilles tendon injury. Runners with AT have altered plantar flexor muscles and such conditions alter their foot strike pattern during running.

## AUTHOR CONTRIBUTIONS

Andere NFB performed the investigation and wrote the original manuscript draft. Godoy-Santos AL and Luna NMS contributed to the conceptualization and funding acquisition. Mochizuki L and Alonso AC contributed to the formal analysis, writing, review and editing of the manuscript. Rodrigues MB, Fernandes TD, and Brech GC supported the investigation, and were responsible for writing, review and editing of the manuscript. Soares-Júnior JM contributed to the formal analysis and manuscript writing and review. Greve JMA supervised and contributed to the writing, review and editing of the manuscript.

## Figures and Tables

**Table 1:** Baseline characteristics of the groups.

	ATG (n=24) Mean (SD)	HRG (n=24) Mean (SD)	NRG (n=24) Mean (SD)
Sex (M/F)	16/8	15/9	11/13
Age (years)	40.5 (7.5)	38.1 (6.4)	35.9 (7.3)*
Body mass (kg)	65.6 (9.5)	67.1 (10.2)	66.8 (10.9)
Height (cm)	170 (0.1)	171 (0.1)	169 (0.1)
Body mass index (kg/m^2^)	22.8 (2.1)	22.9 (2.1)	23.1 (2.1)

ATG: Achilles tendinopathy group, HRG: healthy runners’ group, NRG: non-runners’ group, M: male, F: female, SD: standard deviation.

* Difference between the ATG and NRG after Bonferroni post-hoc test, *p*≤0.05

**Table 2:** Test results for the hypothesis of equality of the means of kinetic variables.

	ATG (n=24) Mean (SD)	HRG (n=24) Mean (SD)	NRG (n=24) Mean (SD)	*p*-value
Fz Max (N. N^-1^)	2.1 (0.4)	2.2 (0.3)	2.0 (0.3)	0.12
Fz Avg (N. N^-1^)	1.2 (0.2)	1.2 (0.1)	1.2 (0.1)	0.24
iSTR (BW.sec)	0.039 (0.01)^a,b^	0.033 (0.01)	0.036 (0.01)	<0.01*
iTOTAL (BW.sec)	0.28 (0.06)	0.28 (0.08)	0.29 (0.06)	0.15

ATG: Achilles tendinopathy group, HRG: healthy runners’ group, NRG: non-runners’ group, Fz Max: maximum force along the vertical axis, Fz Avg: average force along the vertical axis, iSTR: ground reaction force (GRF) integral during the first 50 ms of contact, iTOTAL: GRF integral during the full stance phase. N: newton, BW: body weight, sec: seconds.

^a^significantly different from the HRG and ^b^significantly different from the NRG (analysis of variance and Bonferroni post-hoc test, *p≤0.05).

**Table 3:** Test results for the hypothesis of equality of the means of isokinetic variables at 60°/sec.

	ATG (n=24) Mean (SD)	HRG (n=24) Mean (SD)	NRG (n=24) Mean (SD)	*p-*value
**Concentric mode**				
Plantar flexion peak torque/BW (%)	80.2 (25.0)	81.5 (28.0)	82.6 (22.5)	0.86
Plantar flexion total work (J)	52.0 (14.3)	53.8 (17.6)	56.3 (16.2)	0.51
Dorsiflexion peak torque/BW (%)	35.9 (5.3)	37.8 (5.1)^c^	33.2 (5.4)	<0.01*
Dorsiflexion total work (J)	26.8 (7.2)	30.9 (8.7)	28.5 (8.9)	0.10
**Concentric-eccentric mode**				
Plantar flexion concentric peak torque/BW (%)	179.2 (34.2)	170.8 (48.2)	157.4 (35.9)	0.06
Plantar flexion concentric total work (J)	102.2 (27.5)^c^	96.0 (29.9)	84.4 (22.9)	0.02*
Plantar flexion eccentric peak torque/BW (%)	196.0 (46.1)	199.8 (45.2)	181.9 (38.7)	0.11
Plantar flexion eccentric total work (J)	119.6 (21.6)	123.1 (23.9)	105.7 (23.2)^a,b^	<0.01*

ATG: Achilles tendinopathy group, HRG: healthy runners’ group, NRG: non-runners’ group, BW: body weight, SD: standard deviation.

^a^significantly different from the ATG, ^b^significantly different from the HRG, and ^c^significantly different from the NRG (analysis of variance and Bonferroni post-hoc test, *p≤0.05).

**Table 4:** Test results for the hypothesis of equality of the means of isokinetic variables at 120°/sec.

	ATG (n=24) Mean (SD)	HRG (n=24) Mean (SD)	NRG (n=24) Mean (SD)	*p*-value
**Concentric mode**				
Plantar flexion peak torque/BW (%)	64.5 (18.3)	67.9 (14.2)	61.6 (14.0)	0.16
Plantar flexion total work (J)	172.4 (53.9)^b^	205.8 (47.6)	176.1 (68.7)	0.02*
Dorsiflexion peak torque/BW (%)	27.6 (4.5)	28.6 (4.5)	25.1 (3.3)^a,b^	0.01*
Dorsiflexion total work (J)	63.9 (22.9)	74.5 (20.3)	63.8 (28.2)	0.06
**Concentric/ eccentric mode**				
Plantar flexion concentric peak torque/BW (%)	176.5 (37.1)^c^	170.2 (41.9)	152.4 (32.6)	0.01*
Plantar flexion concentric total work (J)	490.9 (73.8)	461.3 (112.0)	415.2 (148.0)	0.02*
Plantar flexion eccentric peak torque/BW (%)	194.6 (34.7)	186.8 (35.3)	171.6 (33.5)^a,b^	0.01*
Plantar flexion eccentric total work (J)	435.3 (72.4)	439.0 (98.8)	348.2 (72.9)^a,b^	<0.01*

ATG: Achilles tendinopathy group, HRG: healthy runners’ group, NRG: non-runners’ group, BW: body weight, SD: standard deviation.

^a^significantly different from the ATG, ^b^significantly different from the HRG, and ^c^significantly different from the NRG (analysis of variance and Bonferroni post-hoc test, *p≤0.05).
