# Erratum for: Biomechanical evaluation in runners with Achilles tendinopathy

**DOI:** 10.6061/clinics/2021/e2803err

**Published:** 2021-08-16

CLINICS (Sao Paulo). 2021;76:e2803err

**Erratum for: doi: https://doi.org/10.6061/clinics/2021/e2803, published in 2021.**

In the article **Biomechanical evaluation in runners with Achilles tendinopathy**

Replace **“Alexandre Leme Godoy”** and **“Andere NFB, Godoy AL, Mochizuki L, Rodrigues MB, Fernandes TD, Soares-Júnior JM, et al. Biomechanical evaluation in runners with Achilles tendinopathy. Clinics (Sao Paulo). 2021;76:e2803”** for:

**“Alexandre Leme Godoy-Santos”** and **“Andere NFB, Godoy-Santos AL, Mochizuki L, Rodrigues MB, Fernandes TD, Soares-Júnior JM, et al. Biomechanical evaluation in runners with Achilles tendinopathy. Clinics (Sao Paulo). 2021;76:e2803”**

and Page 5 – AUTHOR CONTRIBUTIONS

where it reads **“Godoy AL”** replace for **“Godoy-Santos AL”**

